# An eye-movement database of bilingual language control for Chinese-English bilinguals

**DOI:** 10.1038/s41597-025-04628-2

**Published:** 2025-02-25

**Authors:** Tao Wang, Yue Wang, Haibo Hu, Xing Wang, Shengdong Chen, Yiming Yang

**Affiliations:** 1 School of Psychology, Qufu Normal University, 57 Jing Xuan Road, Qufu, 273165 China; 2 College of Foreign Languages, Qufu Normal University, 57 Jing Xuan Road, Qufu, 273165 China; 3 College of Foreign Languages, Hunan University, No.2 Lushan South Road, Changsha, 410082 China; 4 School of Linguistic Sciences and Arts, Jiangsu Normal University, 57 Heping Road, Xuzhou, 221009 China; 5 Collaborative Innovation Center for Language Ability, Jiangsu Normal University, 57 Heping Road, Xuzhou, 221009 China

**Keywords:** Neurophysiology, Human behaviour

## Abstract

The current absence of an eye-tracking database that explores bilingual language control and how intra-sentence code-switching types influence the language control process limits our deeper understanding of bilingual control mechanisms. To address this issue, we present a database containing eye-movement recordings collected during a silent reading task combined with language switching paradigm. The database contains typical measures of eye movement data of 160 Chinese and their translation equivalent English words from 40 high-proficient and 40 low-proficient participants across 1280 Chinese, English and intra-sentential code-switching sentences. This database enables researchers to test the impacts of both intra-sentential code-switching and the second language proficiency on bilingual language control and the underlying cognitive mechanisms.

## Background & Summary

In recent decades, the surge of globalization has led to an increase in individuals who use two or more languages in their daily life, who are generally called bilinguals^1,2^. When bilinguals speak or comprehend a language, the other language is also activated^3^. Such parallel activation leads to cross-language interference. Therefore, bilinguals need to maintain separation between their two languages to prevent interference and to select one language or the other in a given communicative situation^4,5^. Such cognitive abilities are referred to as bilingual language control^6^, which enables bilinguals to communicate effectively across diverse contexts. Studies on bilingual language control provide valuable insights into the cognitive mechanisms underlying the regulation of two languages within an individual^6^, the impact of bilingual language control on executive functions^7^, and strategies to optimize second language education to enhance students’ bilingual language control^8,9^.

Eye-tracking has been extensively used to study both auditory and written comprehension in psycholinguistic studies, and has emerged as a powerful tool for investigating the complex cognitive processes involved in bilingual language control. Compared with traditional behavioural measures such as reaction time, eye-tracking provides a non-invasive, online, and ecologically valid method of investigating the reading process at the word, sentence, and discourse levels^10^. By leveraging the unique advantages of eye-tracking, researchers can objectively evaluate the online cognitive processes involved in language selection, inhibition^11^, and switching^12^. Specifically, eye-tracking technique can extract objective indicators from different stages of cognitive processing of sentences. For example, the first fixation duration (FFD) can reflect early lexical access^13^, whereas regression-path duration (RPD) is sensitive to the detection of processing difficulty at later stages of sentence processing^14^. Thus, we are able to know how code-switching effect (a classic index of the cognitive control mechanisms) demonstrates at the early and later processing stages. Furthermore, eye-tracking technology can also provide spatial dimensions related to eye movement positions, such as saccade amplitude, fixation count, and skipping rate^15^. Therefore, integrating eye-tracking technology with bilingual control paradigms aids in a deeper understanding of the bilingual control process^16^.

Employing the language switching paradigm, previous studies found the so-called code-switching effect, which typically shows that performance is poorer in trials requiring a switch between languages compared to those with language repetition^17,18^. This effect can be influenced by various factors, including external conditions and individual differences, such as the second language proficiency^19^. Specifically, the code-switching effect in early studies was found by using isolated stimuli such as digits, pictures, and words, lacking a rich linguistic context^20^. Recent studies have found the code-switching effect exist at the sentence level^21,22^ too. This extension from isolated stimuli to sentences reflects deepening understanding of the universal mechanisms involved in bilingual language control. However, the existing eye-tracking studies in the domain of bilingual language control have generally limited their sentential code-switching stimuli to a single intra-sentential code-switching type^16,23,24^, thus ignoring the potential effect of code-switching types on bilingual language control. The current absence of an eye-tracking database that explores bilingual language control and how intra-sentence code-switching types influence the language control process limits our deeper understanding of bilingual control mechanisms. To address this issue, we aim to report an eye-movement database using the silent reading task combined with the language switching paradigm, including the three intra-sentential code-switching conditions of Chinese-English bilinguals.

Specifically, the database includes eye movement data from a classical language switching paradigm^25,26^ combined with eye tracking, in which three types of intra-sentential code-switching sentences and two types of baseline sentences (Chinese an d English) are pseudo-randomly presented (Table 2). All experimental sentences are in the structure of attaching a subordinate clause in front of the main clause. The subject of the main clause is the critical word, at which point the switching between languages occurs. Furthermore, second-language proficiency has been found to be a key factor influencing bilingual language control^27,28^. Therefore, the data of both high- and low-second language proficiency individuals was included in this database.

This database enables researchers to investigate the impacts of both intra-sentential code-switching and second language proficiency on bilingual language control and the underlying cognitive mechanisms. By providing a comprehensive dataset that covers a diverse range of language scenarios and proficiency levels, the present study aims to contribute to a more complete understanding of the complex phenomenon of bilingual language control.

## Methods

### Participants

Data were obtained from 80 undergraduate participants (72 female; *M*_age_ = 21.5, *SD*_age_ = 1.74) during a language switching paradigm from Qufu Normal University, Shandong, China. This study was approved by and performed in accordance with guidelines and regulations of the Institutional Ethics Committee of the Qufu Normal University (approval number: 2024168). All participants were native Chinese speakers with normal or corrected-to-normal vision. Each participant read and signed the informed consent form before the experiment, agreeing with the use of their data for research purpose and allowing data sharing under the condition of maintaining anonymity.

Prior to the experiment, all participants completed a series of assessments to evaluate their second language proficiency and code-switching tendencies. First, they filled out a self-report questionnaire, which asked them to provide their age of acquisition for the first language (Chinese) and age of learning the second language (English). Participants also rated their proficiency in listening, speaking, reading, and writing for both languages on a 5-point scale, with 5 representing the highest level of proficiency. Second, participants completed the Oxford Placement Test (OPT), which served as an objective measure of L2 (English) proficiency^29^. The OPT has a maximum total score of 60 points. To categorize participants into high- and low-proficiency groups, we considered their self-rated language skills, academic major (English major vs. non-English major), and OPT scores. Specifically, participants were classified as high-proficient if they were English majors and scored 50 or above on the OPT. The low-proficient group consisted of non-English majors who scored between 20 and 35 on the OPT. Third, participants filled out the 12-item Bilingual Switching Questionnaire (BSWQ)^30^, which assessed their self-reported frequency of code switching on a 5-point scale. There were totally 40 high-proficient participants and 40 low-proficient participants. The results of self-report questionnaire, OPT and BSWQ of high- and low-proficient groups are presented in Table 1.

**Table 1:** Results of self-report questionnaire, OPT and BSWQ.

		High-proficient		Low-proficient	
		*M*	*SD*	*M*	*SD*
L1	Listening	4.475	0.554	4.452	0.629
	Speaking	4.225	0.620	4.448	0.581
	Reading	4.225	0.620	4.373	0.567
	Writing	3.700	0.564	3.893	0.527
	AoA	0.525	0.506	0.575	0.501
L2	Listening	3.025	0.620	2.288	0.659
	Speaking	3.100	0.632	1.998	0.627
	Reading	3.675	0.474	2.998	0.535
	Writing	3.100	0.379	2.355	0.588
	AoA	8.000	1.051	8.350	1.220
	OPT	52.500	2.736	31.280	3.863
	Code Switching Frequency	32.050	1.974	31.925	2.379

### Materials

The experiment included a total of 1,280 sentences across 8 conditions: 2 non-switch conditions and 6 intra-sentential code-switching conditions, with 160 sentences per condition (see Table 2 for examples). Additionally, 40 filler sentences without code switches were also included in each condition to encourage natural reading. The filler sentences were comparable to the target sentences in terms of length and syntactic complexity, but did not include the same critical lexical items as the experimental sentences.

**Table 2:** The experimental sentences used in the silent reading task.

Types	Language	Definition	Examples
Non-switch	Chinese	All words are Chinese.	,
	English	All words are English.	Upon entering the classroom, those naughty students quieted down immediately.
Insertion	Chinese	The subject noun of the main clause is in Chinese, while the rest of the sentence is in English.	Upon entering the classroom, those naughty quieted down immediately.
	English	The subject noun of the main clause is in English, while the rest of the sentence is in Chinese.	, students
Alternation	Chinese	The part of a sentence before the subject of the main clause is in English, and the remaining part is in Chinese.	Upon entering the classroom, those naughty
	English	The part of a sentence before the subject of the main clause is in Chinese, and the remaining part is in English.	, students quieted down immediately.
Dense code-switching	Chinese	The first noun in the subordinate clause and the subject noun of the main clause are in Chinese, while all other words are in English.	Upon entering the , those naughty quieted down immediately.
	English	The first noun in the subordinate clause and the subject noun of the main clause are in English, while all other words are in Chinese.	classroom, students

*Note*. Chinese, English and code-switching example sentences have the same meaning.

For the intra-sentential code-switching conditions, the sentences were constructed based on the classification system proposed by Muysken^31^, who classified intra-sentential code-switching types into insertion, alternation and dense code-switching, and the standard of sentence construction by Hofweber^26,32^. Specifically, the insertion sentences involve the import of lexical items from an embedded language into a matrix language, which consistently provides the grammatical frame of the bilingual’s utterances^26^. The alternation sentences involve less frequent switching between longer stretches of languages. The dense code-switching sentences involve a switch occurring at the noun in the subordinate clause in addition to a switch within the noun phrase in the main clause^25^.

To control the length of the sentences, the Chinese versions contained 17–22 characters, while the English versions contained 10–14 words. All experimental sentences were structured with a subordinate clause preceding the main clause, with the subject of the main clause serving as the critical word.

To ensure the quality of the sentences, three English-major research assistants first reviewed the grammar and meaning of all sentences. Second, 40 students with English proficiency similar to the low-proficiency group rated the processing difficulty of the sentences on a 7-point scale (1 = extremely easy, 7 = extremely difficult). Result showed that the mean rating scores of all sentences were 2.01 (*SD* = 1.13), suggesting that all sentences are easy to understand.

To mitigate potential carryover effects from repeated exposure to identical semantic content, the experimental stimuli were divided into eight lists using a Latin square design. Each list contained 160 critical code-switching sentences intermixed with 40 filler sentences, resulting in a total of 200 sentences per list. This design ensured that each participant was exposed to a unique set of experimental materials.

Moreover, to encourage attentive reading, 70% of the total sentences were followed by a “Yes/No” comprehension question. The answers were evenly split between “Yes” and “No” responses. For instance, the sentence “Upon entering the classroom, those naughty students quieted down immediately” was followed by the comprehension question “Were students quiet after entering the classroom?” Participants needed to press “F” or “J” to make a “Yes” or “No” response, respectively.

### Data acquisition

Prior to the formal experiment, participants first performed a practice session consisting of 5 sentences followed by a 9-point calibration procedure. Between trial presentations, drift corrections were performed to maintain the accuracy of the eye-tracking data.

During the formal experiment, the participants were asked to read the sentences silently in a natural speed. After finishing reading the sentences, they were instructed to press the space key to answer questions or move into the next trial. If a question sentence appeared, participants needed to press “F” or “J” to make a response. The entire experiment was divided into 4 sessions, with short breaks provided between sessions to allow participants to rest. The formal experiment lasted for about 35 to 40 minutes for the high-proficient group and about 45 to 50 minutes for the low-proficient group.

An SR Research EyeLink 1000 Plus monitored and recorded participants’ eye movements from the right eye at a sampling rate of 1000 Hz while participants read sentences for comprehension. The materials were presented in black Times New Roman font size 14 for English words and in black Song font size 14 for Chinese words on a 19-inch DELL computer screen in a single line. Monitor resolution was 1024 × 768 with a refresh rate of 150 Hz. Participants were seated with their chin resting on a chin rest approximately 60 cm from the monitor.

### Word segmentation

**Fig. 1:**
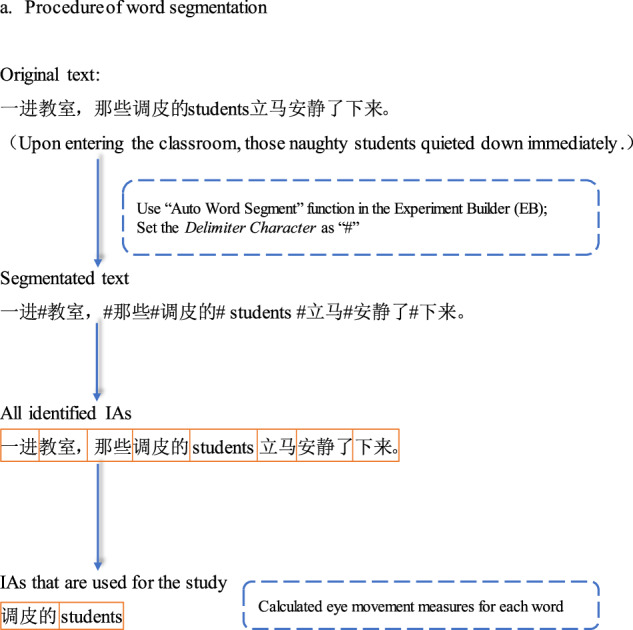
Procedure of word segmentation.

The word segmentation procedure is illustrated in Fig. 1. Given the absence of explicit word boundary markers in the Chinese text, we manually inserted the “#” symbol between words based on common Chinese word usage patterns. For the English words, we placed the “#” symbol between each individual word. This segmentation strategy was then implemented in the “Auto Word Segment” function of the Experiment Builder (EB) software, using the “#” character as the designated delimiter. As shown in Fig. 1, two regions of interest were defined for data analysis: the subject noun in the main clause and the word immediately preceding it. These regions were selected as the primary focus for reporting the study’s findings.

### Pre-processing of eye-movement measures

Before exporting the eye-movement data, fixation durations less than 40 ms or longer than 800 ms were excluded because they could not reflect proper language processing^33^. Meanwhile, the data without fixations were marked as NA. Consequently, 9.98% of the total eye movement data was excluded.

We reported 11 eye-movement measures for the two regions of interest. Table 3 presents the definitions and abbreviations of these measures. In order to help users easily search the measures they are interested in the database, we have divided these 11 eye-movement measures into two categories based on temporal and spatial dimensions^15^. Specifically, the eye-movement measures of temporal dimension are related to when the eyes move, whereas the measures of spatial dimension are related to where the eyes move. The data in the database is arranged in the order of temporal measurements first, followed by spatial measurements, consistent with the sequence presented in Table 3.

**Table 3:** Definitions and Abbreviations of the Nine Eye-Movement Measures.

Eye-Movement Measures		Abbreviations	Definitions
Temporal	First fixation duration	FFD	Duration of the first fixation event that was within the current interest area.
	Second fixation duration	SFD	Duration of the second fixation in the current interest area, regardless of run.
	Gaze duration	GD	Dwell time of the first run (i.e., the sum of the duration of all fixations in the first run of fixations within the current interest area).
	Regression path duration	RPD	The amount of time beginning with the first fixation on the critical stimulus until the eyes cross the right-hand boundary of the region
	Total dwell time	TDT	The sum of the duration across all fixations that fell in the current interest area.
Spatial	Saccade amplitude	SA	Amplitude (in degrees of visual angle) of the first saccade entering into the current interest area.
	Fixation count	FC	Total number of fixations falling in the interest area.
	Skip	PS1	An interest area is considered skipped (i.e., IA_SKIP = 1) if no fixation occurred in first-pass reading.
	First run fixation count	FFC	Number of all fixations in a trial falling in the first run of the current interest area.
	Regression in count	RI	Number of times interest area was entered from later parts of the sentence.
	Regression out count	RO	Number of times interest area was exited to the earlier parts of the sentence before leaving the current interest area in a forward direction.

## Data Records

The database is freely available on OSF repository^34^ under the CC BY 4.0 License. The data of interest areas are provided in the file “IA_data.xlsx” and the materials of the experiment are provided in the file “Sentences.xlsx”.

All the data mentioned above are available on the website https://osf.io/8j9uv/. The file named “IA_data.xlsx” contains the raw data of interest areas exported from Data Viewer, a data analysing software developed by SR Research. In this file, each row provides information and data of the interest area observed by a subject during reading. The eighteen columns provide the following information.

RECORDING_SESSION_LABEL: Label of the data file of participants.
L2 PROFICIENCY: Participants’ English proficiency (L and H represents Low and High, respectively).
TRIAL_INDEX: Sequential order of the trial in the recording (from 1 to 160).
4.TRIAL_LABEL: Label of the trial, unique number for the sentences.
CONFITION: The specific condition for each sentence (from condition1 to 8).
IA_ID: Ordinal ID of the current interest area.
IA_LABEL: Label for the current interest area, the visual form of each word for which the eye-movement measures are calculated.
IA_FIRST_FIXATION_DURATION: provides the FFD for each word.
IA_SECOND_FIXATION_DURATION: provides the SFD for each word.
IA_FIRST_RUN_DWELL_TIME: provides the GD for each word.
IA_REGRESSION_PATH_DURATION: provides the RPD for each word.
IA_DWELL_TIME: provides the TDT for each word.
IA_FIRST_SACCADE_AMPLITUDE: provides the SA for each word.
IA_FIXATION_COUNT: provides the FC for each word.
IA_SKIP: provides the PS1 for each word.
IA_FIRST_RUN_FIXATION_COUNT: provides the FFC for each word.
IA_REGRESSION_IN_COUNT: provides the RI for each word.
IA_REGRESSION_OUT_COUNT: provides the RO for each word.

All sentences used for the bilingual control experiment are available in the file named “Sentences.xlsx”. In this file, each row provides information for a sentence. The four columns provide the following information.

1. TRIAL_LABEL: Label of the trial, unique number for the sentences.
2. CONFITION: The specific condition for each sentence (from condition1 to 8).
3. LIST: The list of materials to which a sentence belongs.
4. SENTENCES: The visual form of each sentence.

## Technical Validation

### Qualitative validation

The validity of the bilingual language control dataset was evaluated using a multi-pronged approach. First, the experimental sentences were carefully constructed and reviewed by three bilingual experts to ensure that these sentences met the target criteria for length, syntactic complexity and structure consistency, and lexical properties. Second, the data were collected in the same laboratory using the same procedures and tasks (i.e., silent reading task). Third, eye-movement measures were all generated and exported from the EyeLink Data Viewer. Consistent with previous research^35–37^, all fixation durations shorter than 40 ms or longer than 800 ms were excluded before exporting the data.

### Quantitative validation

To quantitatively validate the database, we replicated well-established effect of switching language on three key eye-tracking measures: first fixation duration (FFD), gaze duration (GD), and regression path duration (RPD) for the critical words. The code-switching effect, defined as the performance difference between switch trials and non-switch trials, has been widely used as a classic index of the cognitive control mechanisms involved in bilingual language processing^3,25,35,38–40^. To verify whether the current database can effectively investigate bilingual language control, we used R software to analyse the code-switching effect (results are shown in Table 4). Switched target words showed shorter FFDs, shorter GDs (though not significant), and shorter RPDs compared to non-switched target words when switching from the L1 (Chinese) to the L2 (English). Conversely, when switching from the L2 to the L1, switched target words exhibited shorter FFDs, shorter GDs, but longer RPDs than non-switched target words. These results align with the existing bilingual language control literature, providing evidence for the reliability and validity of the current eye-movement database.

**Tabel 4:** Results for the Effect of Code Switching on Three Primary Eye-Movement Measures.

Measures	Direction	*M* (*SD*)		*t* value
		Switched	Non-switched	
FFD	L1 → L2	258.96 (102.73)	293.59 (119.76)	10.37**
	L2 → L1	219.98 (77.11)	228.42 (80.87)	3.66**
GD	L1 → L2	589.59 (454.14)	592.71 (399.71)	0.26
	L2 → L1	296.51 (163.28)	313.01 (183.48)	3.20**
RPD	L1 → L2	884.77 (636.07)	1051.30 (834.21)	7.3086**
	L2 → L1	531.89 (386.99)	472.46 (312.70)	−6.1856**

*Note*. * *p* < 0.05, **p < 0.001. FFD, first fixation duration; GD, gaze duration; TDT, total dwell time.

## Data Availability

The data pre-processing and eye-movement measure calculating were conducted with EyeLink DataViewer. Two R scripts (“Descriptions.R”, “Technical_validation.R”) resulting from descriptive statistics and technical validation has been released in the repository of OSF^34^ (https://osf.io/8j9uv/).
